# Incidence, household transmission, and neutralizing antibody seroprevalence of Coronavirus Disease 2019 in Egypt: Results of a community-based cohort

**DOI:** 10.1371/journal.ppat.1009413

**Published:** 2021-03-11

**Authors:** Mokhtar R. Gomaa, Amira S. El Rifay, Mahmoud Shehata, Ahmed Kandeil, Mina Nabil Kamel, Mohamed A. Marouf, Mohamed GabAllah, Ahmed El Taweel, Ahmed E. Kayed, Omnia Kutkat, Yassmin Moatasim, Sara H. Mahmoud, Noura M. Abo Shama, Mohamed El Sayes, Ahmed Mostafa, Rabeh El-Shesheny, Pamela P. McKenzie, Richard J. Webby, Ghazi Kayali, Mohamed A. Ali

**Affiliations:** 1 Center of Scientific Excellence for Influenza Viruses, National Research Centre, Giza, Egypt; 2 Department of Infectious Diseases, St. Jude Children’s Research Hospital, Memphis, Tennessee, United States of America; 3 Department of Epidemiology, Human Genetics, and Environmental Sciences, University of Texas, Houston, Texas, United States of America; 4 Human Link, Dubai, United Arab Emirates; Erasmus Medical Center, NETHERLANDS

## Abstract

SARS-CoV-2 virus is transmitted in closed settings to people in contact with COVID-19 patients such as healthcare workers and household contacts. However, household person-to-person transmission studies are limited. Households participating in an ongoing cohort study of influenza incidence and prevalence in rural Egypt were followed. Baseline enrollment was done from August 2015 to March 2017. The study protocol was amended in April 2020 to allow COVID-19 incidence and seroprevalence studies. A total of 290 households including 1598 participants were enrolled and followed from April to October 2020 in four study sites. When a participant showed respiratory illness symptoms, a serum sample and a nasal and an oropharyngeal swab were obtained. Swabs were tested by RT-PCR for SARS-CoV-2 infection. If positive, the subject was followed and swabs collected on days three, six, nine, and 14 after the first swab day and a serum sample obtained on day 14. All subjects residing with the index case were swabbed following the same sampling schedule. Sera were collected from cohort participants in October 2020 to assess seroprevalence. Swabs were tested by RT-PCR. Sera were tested by Microneutralization Assay to measure the neutralizing antibody titer. Incidence of COVID-19, household secondary attack rate, and seroprevalence in the cohort were determined. The incidence of COVID-19 was 6.9% and the household secondary attack rate was 89.8%. Transmission within households occurred within two-days of confirming the index case. Infections were asymptomatic or mild with symptoms resolving within 10 days. The majority developed a neutralizing antibody titer by day 14 post onset. The overall seroprevalence among cohort participants was 34.8%. These results suggest that within-household transmission is high in Egypt. Asymptomatic or mild illness is common. Most infections seroconvert and have a durable neutralizing antibody titer.

## Author summary

SARS-CoV-2 virus is transmitted via close contact with infected persons, airborne droplets, and contaminated surfaces. People in closed settings with COVID-19 patients, such as healthcare workers and household contacts, were more likely to become infected. Egypt, like all other countries, is currently facing the COVID-19 pandemic. However, data on incidence and seroprevalence in this country is lacking. We followed 290 households participating in an ongoing cohort study of influenza and coronavirus incidence and prevalence. The incidence of COVID-19 was 6.9% and the household secondary attack rate was 89.8%. Transmission within households occurred within two-days of confirming the index case. Infections were asymptomatic or mild with symptoms resolving within 10 days. The majority developed a neutralizing antibody titer by day 14 post onset. The overall seroprevalence among cohort participants was 34.8%. The majority of subjects with three months apart paired-samples continued to be seropositive. Within-household transmission is high. Asymptomatic or mild illness is common. Most infections seroconvert and have a durable neutralizing antibody titer. Increasing awareness among the general public about proper ways of dealing with infections within the household may contribute to decreasing the spread in Egypt and areas with similar cultural background and population structure.

## Introduction

The Severe Acute Respiratory Syndrome Coronavirus 2 (SARS-CoV-2) that causes the coronavirus disease 2019 (COVID-19) is now a pandemic and a global crisis. Since 2003, three coronaviruses, the Severe Acute Respiratory Syndrome Coronavirus (SARS-CoV) in 2002–2003, the Middle East Respiratory Syndrome Coronavirus (MERS-CoV) in 2012, and SARS-CoV-2 (2019) emerged to infect human populations causing severe respiratory infections leading to death [1–3].

Since the first detection of SARS-CoV-2 in Wuhan, China, in December 2019, more than 105 million infected cases were reported including over 2.3 million confirmed deaths as of 9 February 2021 [4]. Old age, having chronic diseases such as diabetes, cardiovascular disorders, chronic respiratory illness, hypertension, cancer, and being a health care worker were reported as risk factors to increasing case fatality rate [5].

In Egypt, since the first travel-related case was announced on 14 February 2020, the cumulative infected cases reached more than 120,000 with more than 7,000 deaths [6]. Several interventions were implemented by the Egyptian authorities such as recommending wearing masks, social distancing, isolation of confirmed cases, quarantine of suspected cases and exposed people, personal hygiene, and closure of schools and airports. However, cases continued to be reported. The number of cases peaked between May and July 2020 with the observation of religious and cultural celebrations involving extended family gatherings within households associated with the Holy Month of Ramadan and Fitr Islamic Holiday. The Egyptian Ministry of Health shifted to household isolation for mild cases in May 2020 as compared to hospital isolation for all cases practiced earlier. By July, the number of cases started to decrease and several restrictions were lifted. Case counts remained low but started to increase in November yet remained below 500 cases per day (S1 Fig). Cases concentrated in greater Cairo and other cities but were also reported in rural areas across the country.

The virus is transmitted via close contact with infected persons, airborne droplets, and contaminated surfaces [7,8]. People in closed settings with COVID-19 patients, such as healthcare workers and household contacts, were more likely to become infected [5,9]. Evidence suggested that symptomatic people may spread COVID-19 through direct close contact among individuals rather than asymptomatic carriers [10]. Transmission studies conducted in China [11–14] and the USA [15,16] confirmed the transmissibility within households from primary to secondary cases. Yet, SARS-CoV-2 household person-to-person transmission studies are limited. Here, we followed 290 households participating in an ongoing cohort study of influenza and coronavirus incidence and prevalence. We show the clinical features, household secondary attack rate, and seroconversion among household residents.

## Results

### Incidence and household transmission

As of April 2020, a total of 1598 participants in 290 households at four study sites (Gharbiyah, Kafr El Sheikh, Qalyubiyah, and Fayyoum) were followed up. The median household size was five individuals (range 1–19). The demographic characteristics of those participants are shown in S1 Table. The mean age of the participants was 24 years and the median was 19 years. Around 44% were female and more than half were single/never married. Around a third did not report receiving any formal education, a third completed elementary school, 16% completed intermediate schooling, 6.5% completed schooling, and 9.7% attended college. Around 7% reported smoking, 2% had chronic breathing problems, and 10% reported having non-respiratory chronic diseases.

**Fig 1:**
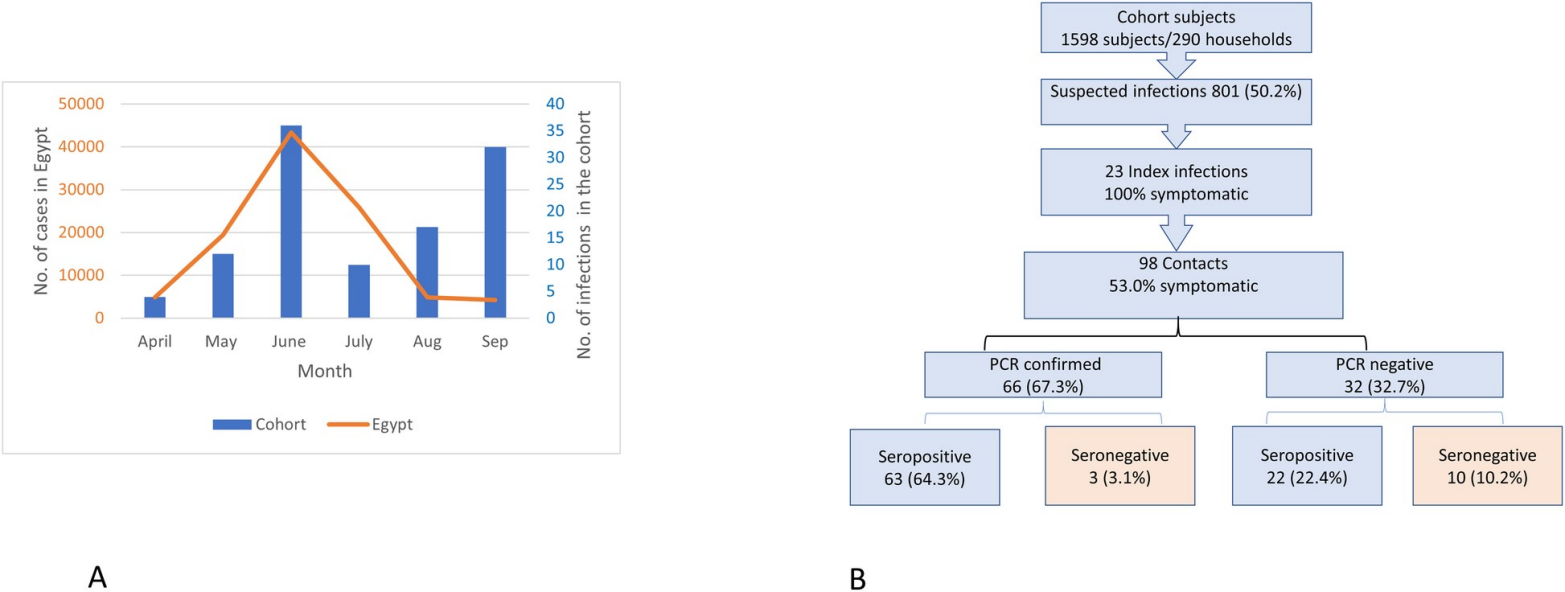
A: Number of infections by month in the cohort and in Egypt. B: Suspected, index, and secondary infections of COVID-19 in the cohort.

Fig 1A shows the monthly reported infections among cohort participants between April and September 2020. Most infections (n = 36) were detected in June but decreased in July and started increasing again in August and September. Additionally, two and three influenza A infections were detected in August and September, respectively.

Fig 1B summarizes the incidence of COVID-19 in the cohort. By the end of September 2020, a total of 801 participants (50.1%) developed clinical criteria that met our COVID-19 case definition (suspected infections). Of those, 23 subjects from 23 different households were confirmed by Real-time reverse-transcription polymerase chain reaction (RT-PCR) to be infected with SARS-CoV-2 and were hence considered index infections. Those index infections had 98 household contacts (range of 2–14 contacts per household) of whom 52 (53%) developed symptoms. Of the contacts, 66 (67%) were RT-PCR confirmed to be infected and 63 of them seroconverted. Twenty-two of the RT-PCR negative contacts seroconverted. Thus, the total number of contact infections was 88 (66 RT-PCR confirmed and 22 seroconversions) and the total number of COVID-19 infections in the cohort was 111 (23 index infections and 88 contacts) (S2 Fig). The overall incidence rate in the cohort for the period April-September 2020 was 6.9% (95% confidence interval (CI): 5.8–8.3), the infection rate among suspected infections was 13.8% (95% CI: 11.6–16.4), and the household secondary attack rate was 89.8% (95% CI: 82.2–94.3). Demographic and general health characteristics of household infections did not statistically differ from non-infected cohort participants.

**Fig 2:**
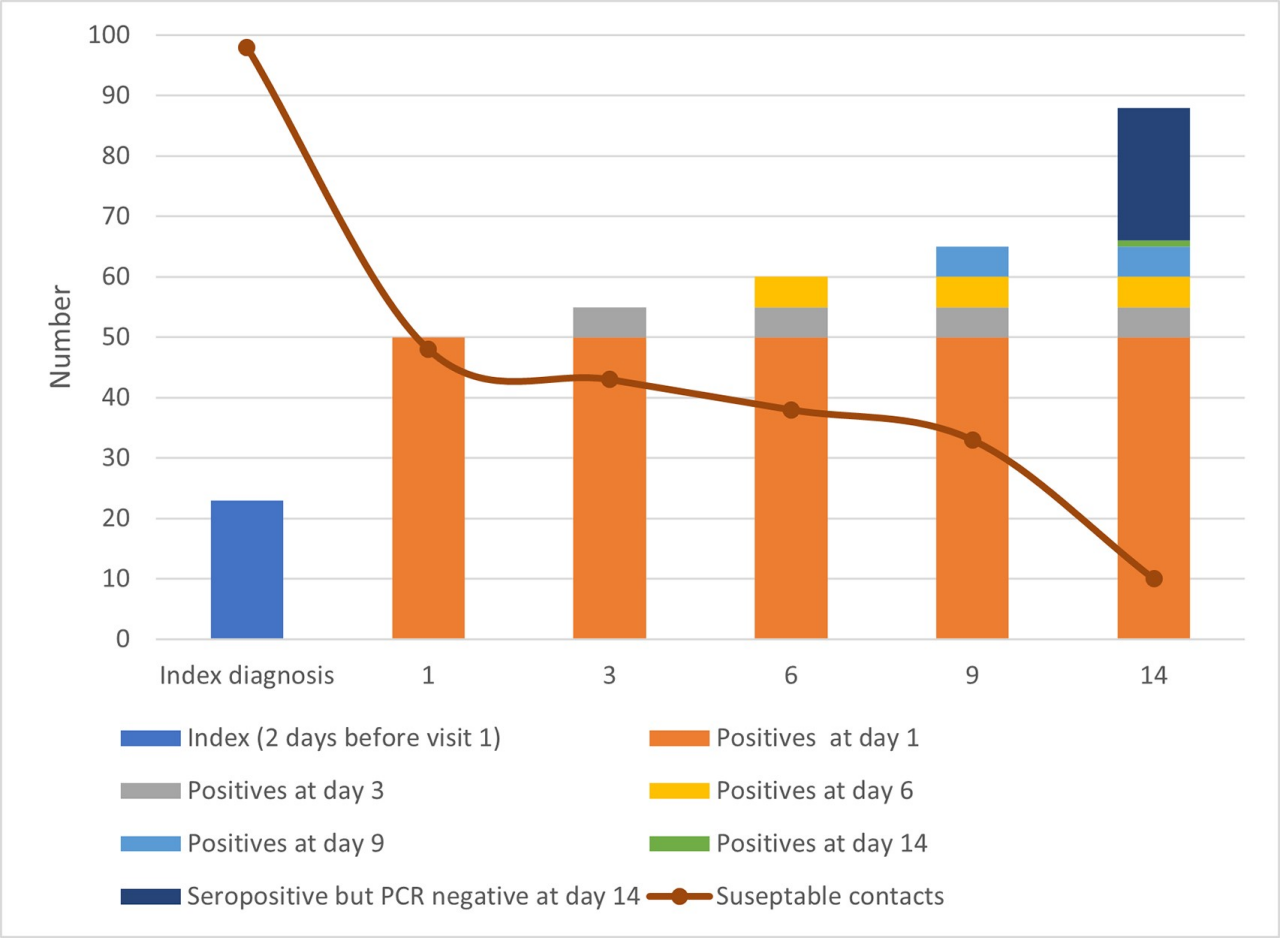
Transmission of COVID-19 infection among close contact households. Except for the dark blue legend, legends indicate the day for the first PCR positive test.

Transmission within household from index to contact infections was mostly within two days after confirmation of index case infection as 50 of the 88 contacts were RT-PCR positive at the first day of sampling (Fig 2). Five additional contacts became RT-PCR on day three, six, and nine of sampling. Only one contact became RT-PCR positive on day 14. The median age of index infections was 33 years (range 11–71 years) while the median age of the infected contacts was 22 years (range 1–80 years). The age difference between infected index and contact infections was not statistically significant. Among the index infections, 14 (60.9%) were females compared to 44 (50%) females among infected contacts (p-value >0.05).

All index infections had symptoms as compared to 52 contacts (Table 1). The most reported symptoms (>70%) among index infections were fever, cough, fatigue, muscle/body aches, headache, and loss of taste/smell. Muscle or body aches was the most commonly-reported symptom among contacts. Most symptoms resolved by day 10 post onset except for loss of taste or smell that lasted a median of 14 days among index infections. In general, contacts had fewer symptoms and had their illness resolved quicker than index infections. All infections were mild not requiring hospitalization.

**Table 1:** Occurrence and duration of symptoms among index and contact infections.

Symptoms	Index infections (n = 23)		Contact infections (n = 52)	
	No. (%)	Median no. of days (range)	No. (%)	Median no. of days (range)
**Fever**	22 (95.6)	4 (1–18)	32 (61.5)	3 (1–14)
**Cough**	18 (78.3)	8 (2–25)	31 (59.6)	7 (2–28)
**Shortness of breath**	14 (60.9)	6 (1–21)	19 (36.5)	3 (1–25)
**Fatigue**	20 (87.0)	7 (2–25)	31 (59.6)	7 (2–30)
**Muscle/Body aches**	21 (91.3)	8 (2–28)	39 (75.0)	7 (3–22)
**Headache**	18 (78.3)	5 (2–11)	30 (57.7)	4 (1–22)
**Loss of taste/smell**	20 (87.0)	14 (3–26)	28 (53.8)	10 (2–30)
**Sore throat**	16 (69.6)	5 (1–14)	31 (59.6)	4 (1–10)
**Congestion/Runny nose**	9 (39.1)	7 (3–14)	23 (44.2)	3 (1–15)
**Nausea/Vomiting**	12 (52.2)	7 (2–14)	13 (25.0)	4.5 (1–11)
**Diarrhea**	11 (47.8)	3 (1–8)	18 (34.6)	3 (1–7)
**Blurred vision**	8 (34.8)	4 (3–10)	11 (21.2)	4 (1–6)
**Anorexia**	16 (69.6)	9 (2–28)	22 (42.3)	7 (2–30)

**Fig 3:**
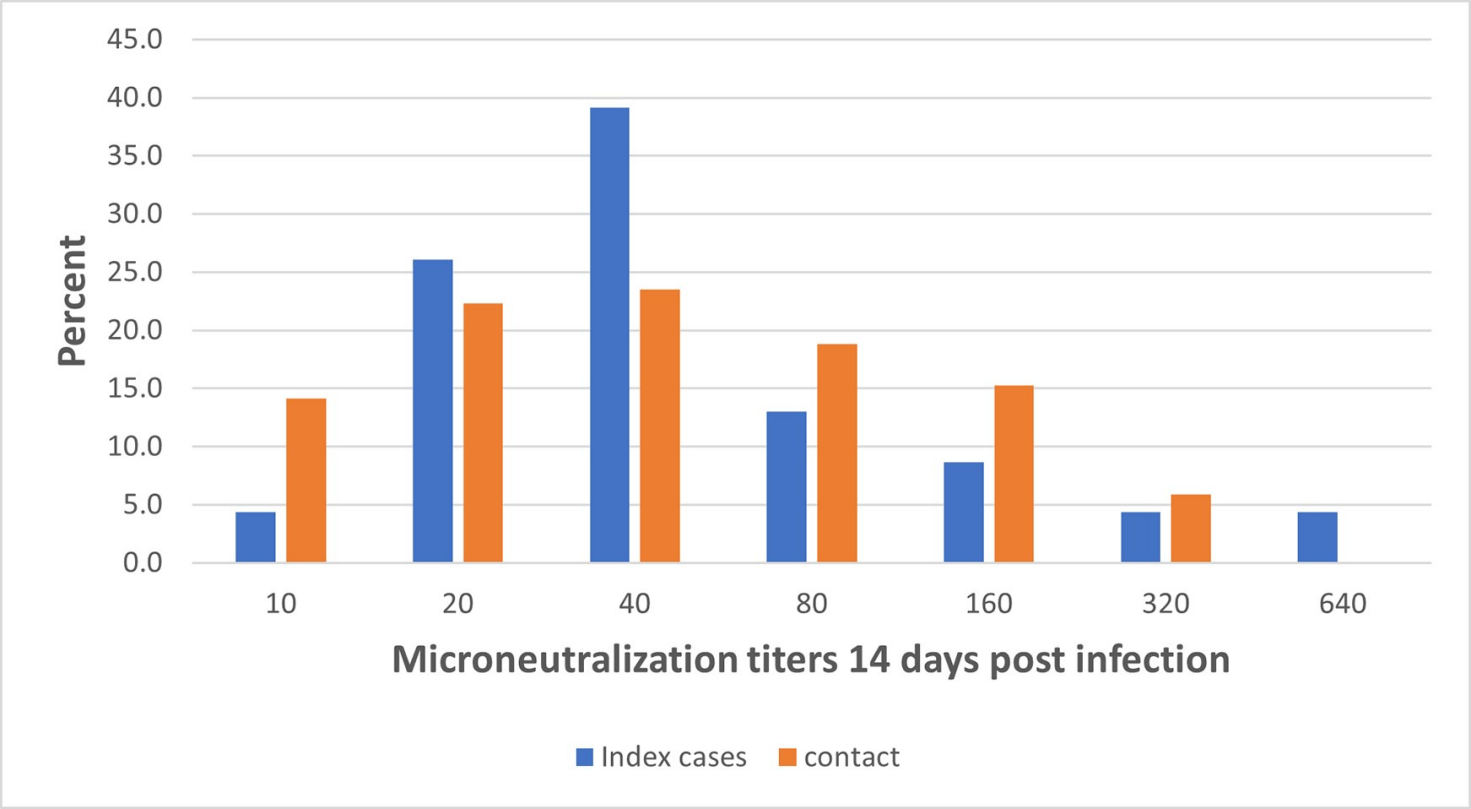
Distribution of antibody titers among infected index and contact infections 14 days post infection.

By day 14 post initial RT-PCR confirmation, all index infections had a neutralizing antibody (nAb) titer (geometric mean titer (GMT) 49.4). Seven (30.4%) had a titer ≥ 1:80. Among the 88 infected contacts, 85 (96.6%) had an antibody titer (GMT 45.3) of whom 34 (38.7%) had a titer ≥ 1:80. The distribution of titers is shown in Fig 3. Three of the index infections had a nAb titer at day 1 of sampling that increased by day 14. Similarly, nine of the contact infections had a titer at day 1 that either increased or remained within 1-fold by day 14 (S2 Table).

### Cohort seroprevalence

All 212 sera collected in April 2020 tested negative by Microneutralization Assay (MN). Of those, 193 individuals were re-sampled in July and 30 of them sero-converted. The distribution of titers among the subjects who were tested in both April and July is shown in S3 Fig. Around 85% of those remained seronegative, 13.5% developed titers between 1:10 and 1:80, and 2% developed titers of 1:160 and above.

**Fig 4:**
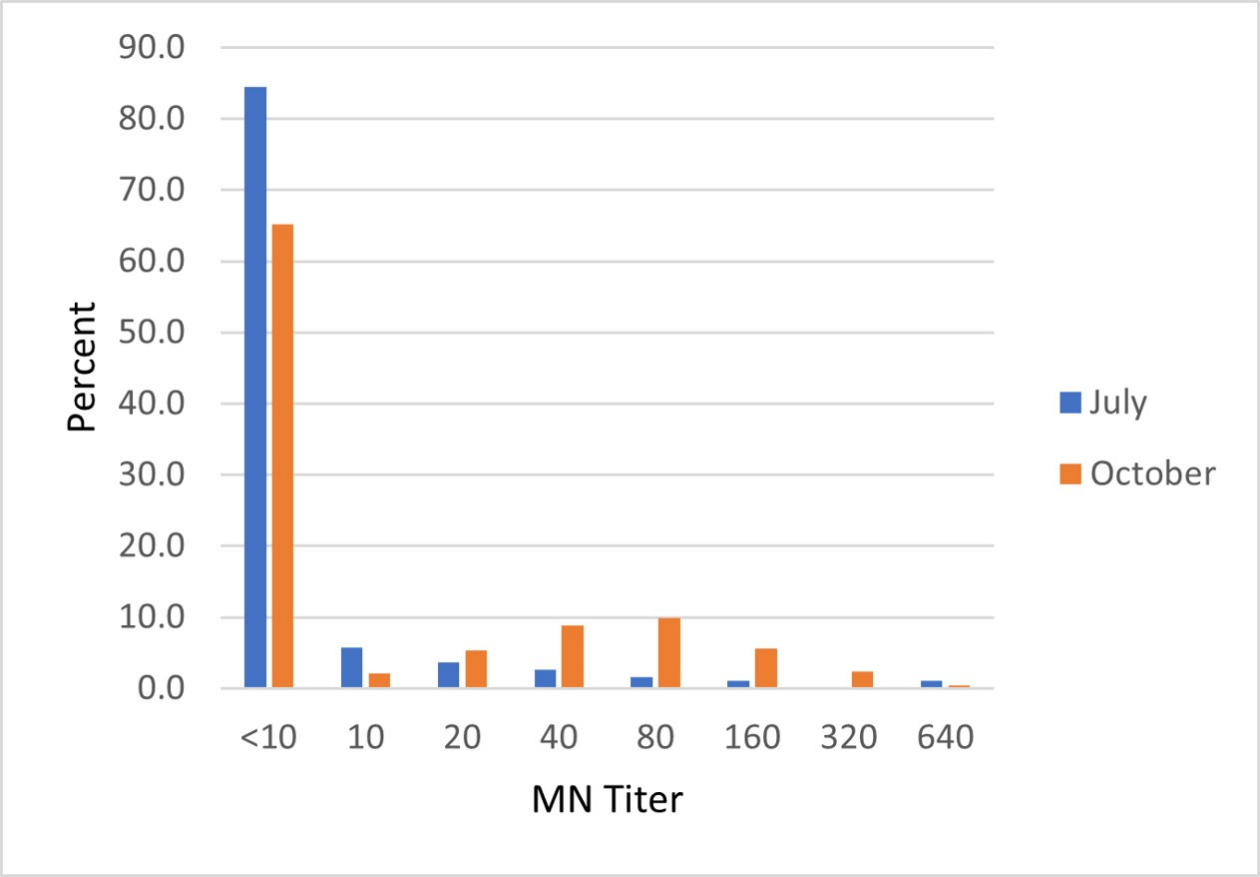
Distribution of neutralizing antibodies among cohort participants, July and October 2020.

The distribution of titers in sera collected in July 2020 is shown in Fig 4. Around 80% were sero-negative, 18% had titers between 1:10 and 1:80, and 2% developed titers of 1:160 and above. The overall GMT was 1:6.9. Age was significantly associated with antibody levels (p-value = 0.017). The mean age of sero-negative participants was 29 years, 27 years for those with antibody titers between 1:80 and 1:160, and 40 years for those with titers greater than 1:160. None of the other variables was associated with antibody titers.

A total of 1260 sera were collected from cohort participants in October 2020. Of those, 438 (34.8%) were seropositive (95% CI: 32.2–37.4) (GMT 61.2). Only 238 (18.5%) participants had a titer ≥ 1:80. The distribution of titers is shown in Fig 4. Of the females, 37.2% were positive as compared to 32.6% of the males (p-value >0.05). There was no statistically significant difference between the ages of the seropositive and seronegative participants.

Of participants who were seropositive in July 2020, 200 were resampled in October 2020. Among those, 69 (34.5%) had a stable titer (within 1-fold change) and 76 (38.0%) had an increase in titer (2 or more folds). Four had a decrease in titer (2 or more folds) but remained seropositive while 51 (25.5%) became seronegative. Titer variation among those 200 subjects is shown in S4 Fig.

## Discussion

This paper is among the few that describe household transmission of COVID-19 and that show seroconversion and neutralizing antibody titers within households in a community rather than clinical settings. Furthermore, the results presented here are the firsts from a cohort study conducted in low-income country.

The six-month incidence of COVID-19 in the cohort was 6.9%. The monthly distribution of new infections matched the distribution of cases in the general Egyptian population for the period April-July 2020 with cases peaking in June then decreasing in July. In Egypt, the first few cases were in March 2020 when the virus was introduced. By June-2020, COVID-19 infections surged potentially due to social activities linked to the last week of the Holy month of Ramadan and Eid-El-Fitr (the religious holiday following Ramadan). Cohort infections increased again in August and September while the number of cases reported in the general Egyptian population remained low. The increase in August coincides with another religious holiday Eid-El-Adha. This difference in incidence between the cohort and the general population may be due to testing only severe cases in the general population compared to testing all symptomatic infections and household contacts in the cohort. Hence, it is likely that the true incidence of disease in Egypt is close to that measured in the cohort. Almost half of the cohort participants reported a related symptom, a relatively high proportion given the time period of the study that is out of the season when respiratory diseases are spread. This may be due to the pandemic scare leading the subjects to become overzealous in self-reporting symptoms.

The household secondary attack rate in our study was more than 89% and is higher than rates reported in other studies. In a study from Wuhan, China, the secondary attack rate was 30% [12]. A similar rate (32%) was calculated in a study in Zhuhai, China [11]. Lower rates were estimated for China in other studies [13,17]. In the USA, secondary attack rates were estimated between 0.5–53% [15,16,18,19]. In Canada, a 14.7% secondary infection rate was calculated [20]. A meta-analysis estimated the rate to be around 17% [21]. Our higher rate may be explained by behavioral factors or by household size. Residents of households of our study may not have isolated themselves from index infections or may not have used proper personal protective equipment as recommended by public health authorities. The median household size in our study was five, potentially larger than that in China or North America, with several individuals sharing same bedrooms. Using protective equipment was shown to reduce secondary attack rates in household settings [22]. Some of the contacts became RT-PCR positive by day 9 or day 14 and hence may potentially be tertiary rather than secondary infections. If those were to be excluded from being secondary, then the secondary attack rate is reduced to 83.7%. Furthermore, there were four contacts that were seropositive at day 0 and did not have an increase in their antibody titer and may have not been related to the household transmission event. If those were excluded, the household secondary attack rate becomes 79.6%. Nonetheless, the household secondary attack rate in our cohort remains high.

Most secondary infections occurred within two days of confirming the index case. This may not be indicative of the true incubation period as the index case was tested when symptomatic. About a third of the secondary infections did not have a positive RT-PCR test probably due to low viral load at the time of sampling or due to improper sampling.

Asymptomatic infection was observed in our study as was detected in other studies with more than 40% of the confirmed secondary infections not showing symptoms [23]. A wide range of symptoms was observed including respiratory and gastro-intestinal manifestations. All the infections had relatively mild disease with symptoms resolving in less than 10 days. Only loss of smell or taste lasted for a median of 14 days. Overall, disease among secondary infections appears to be milder than among index infections with less symptoms reported and shorter duration of illness.

Our data indicate that the majority of the infections we followed (70%) developed nAb titers by day 14. Titers ranged from 1:10 to 1:640. Due to the short time period of follow-up (14 days), it is plausible that detected titers may increase over time and seroconversion may occur in infections that did not seroconvert by day 14. Twelve of the infections we followed had a detectable antibody titer at day of onset. Of those, eight were PCR-confirmed to be infected and eight became symptomatic. The person who did not have a positive PCR did not show any symptoms and had a titer of 1:160 and this titer did not increase over time. This suggests that low-level antibody titers may not fully protect against COVID-19 infection. An alternative explanation is that the detected antibody titer may have been elicited due to infection that has been ongoing before our follow-up commenced or that those infections may have been asymptomatic index infections. Indeed, most of the infections that had a titer at day 1 either stayed within 1-fold or increased by day 14 indicating a progressive antibody response.

Our cohort-wide serological testing revealed that a third of participants were seropositive. This rate increased from zero in April to 20% in July to above 30% in October. This shows that infection is more common than what we detected suggesting that very mild or asymptomatic infections occur frequently. Furthermore, the majority of subjects who were positive three months before the sampling conducted in October continued to have a detectable antibody titer suggesting the durability of neutralizing antibodies over time as indicated by other studies [24]. In a sero-survey among blood donors in Brazil, anti-SARS-CoV-2 antibodies were detected in 4% of the samples [25]. In another population-based study in Brazil, the seroprevalence rate was 0.05% [26]. In the USA, seroprevalence in Los Angeles county was estimated at 4.7% [27]. In a larger study involving several states, calculated seroprevalence rates ranged between 1% and 6.9% [28]. In a population-based study in Geneva, Switzerland, 4.8% of the participants had anti-SARS-CoV-2 IgG [29]. The largest seroprevalence study was conducted in Spain among more than 61,000 participants. Seropositivity was around 5% [30].

In comparison to the above-mentioned reports, our study is the only one that relied on neutralizing antibodies to determine seroprevalence of SARS-CoV-2 antibodies. This could be explained by the presence of COVID-19 case clusters in the villages where the subjects were sampled. However, the measured titers are relatively low as only a small percentage of tested sera had antibody titers greater than 1:160. This could potentially indicate that the majority of those who sero-converted had asymptomatic or mild disease [31]. Another explanation may be related to the fact that we only considered serum dilutions that totally protected the cells from CPE as positive.

Relying on the presence of symptoms in an index case to study within-household transmission is a limitation for this study especially with the commonality of having very mild and asymptomatic COVID-19 patients. This could have led to an underestimation of the calculated overall incidence within the cohort. Additionally, the calculated household secondary attack rates may be biased if the missed asymptomatic index infections did not contribute as much as symptomatic index infections to household transmission. Another limitation is that some contacts may have been asymptomatically infected when the index infection was confirmed hence not meeting the definition of a secondary infection. Data from this study may not be generalizable to the general Egyptian population due to the small sample size and geographic distribution of households. By design, However, our data strongly indicate that household transmission is playing an important role in the spread of COVID-19 in Egypt. It has been previously shown that COVID-19 household transmission and superspreading events may be the main drivers of disease spread [32,33]. Increasing awareness among the general public about proper ways of dealing with cases within the household may contribute to decreasing the spread in Egypt and areas with similar cultural background and population structure.

## Materials and methods

### Ethics statement

Ethical approval for the study was granted by the IRBs of St. Jude Children’s Research Hospital (USA) (reference number 007079, dated March 20, 2020) and Human Link (Lebanon) (dated March 23, 2020) as well as the Research Ethics Committee of the National Research Centre (Egypt) (protocol number 14 155, dated March 22, 2020). Written informed consent was obtained from all subjects over 18 years old, written assent was obtained for children between 14 and 17 years old, parental written consent was obtained for all participants less than 18 years old.

### Cohort study design

Details of the study design and protocol have been previously published [34]. Briefly, households raising backyard poultry were selected from five villages in four Nile Delta governorates (Sharkiyah, Gharbiyah, Kafr El Sheikh, and Qalyubiyah) and Fayyoum governorate starting August 2015. All individuals within the household who were older than two years were invited to participate. Baseline enrollment was completed in March 2017. A total of 2402 subjects were enrolled from 390 households in the five study sites. The study protocol was amended in April 2020, to allow COVID-19 incidence and seroprevalence studies in four study sites (Fayyoum, Gharbiyah, Kafr El Sheikh, and Qalyubiyah) comprising 290 households.

To determine infection rates, study staff visited enrolled households on a weekly basis to check whether any study participant was reporting respiratory illness symptoms of fever of 38°C or higher, cough, sore throat, or shortness of breath. When a study participant was verified to have symptoms, a serum sample was collected, and a nasal swab and an oropharyngeal swab were obtained and tested by RT-PCR for SARS-CoV-2 infection. If any of the swabs tested positive, the subject was considered an index infection, and the study team obtained nasal and oropharyngeal swabs from the participant on days three, six, nine, and 14 after the first swab day as well as a serum sample on day 14. Furthermore, all previously enrolled subjects residing with the index case were swabbed and blood samples obtained following the same sampling schedule two days after the diagnosis of the index case. A symptoms diary was also started for the index infection and all subjects within the household, symptoms data were collected by the study staff.

To determine cohort seroprevalence, sera were collected from 212 subjects in April 2020, 1,244 subjects in July, and 1,260 subjects in October to assess seroprevalence among participants.

Study staff interacted with cohort subjects following strict biosafety procedures including wearing N95 masks, gloves, disposable gowns, and hair and shoe covers. Disposed personal protective equipment were placed in biohazard bags and transported to the lab for proper disinfection and disposal. All staff were rigorously trained on all aspects of study protocol and biosecurity measures.

### Viral testing

Swab samples were subjected to viral RNA extraction using QIAamp Viral-RNA Kit (Qiagen, Germany) according to manufacturer’s instructions. RT-PCR screening assays (E gene, N gene, ORF1b-nsp14, and RdRp gene assays) with gene specific primers and probes [35,36] were conducted using Verso 1-step qRT-PCR Kit (Thermo, USA). A 25 μl total reaction included 5 μl template RNA, 12.5 μl 2x one-step buffer, 1 μl of each forward and reverse primers (10 μM), 0.5 μl probe (10 μM), 1.25 μl RT-Enhancer, 0.25 μl enzyme mixture, and 3.5 μl ddH_2_O. Thermal cycling started with 15 min at 50°C (reverse transcription), 95°C for 15 min (polymerase activation), 45 cycles at 95°C for 15 s (denaturation), then 60°C for 30 s (annealing and extension). The positive control was a synthetic plasmid designed in-house including selected nucleotides that are detected by all used assays.

### Serological testing

MN was conducted to measure the nAb titer in human sera using Vero-E6 (ATCC, CRL-1586) cell monolayers using SARS-CoV-2/Egypt/NRC-03/2020 under biological safety level 3 [37]. This virus was isolated from a swab of a confirmed patient in Egypt (passage 0) and cultured again in Vero-E6 cells to increase titers (passage 1). Stocks used for the MN assay were from passage 2 of the virus. Briefly, the collected sera were inactivated at 56°C for 1 hr. Sera were serially diluted two-fold from 1:10 to 1:1280 in DMEM media supplemented with 4% BSA, 1% antibiotic antimycotic (Gibco, USA), then mixed with equal volume of 100 tissue culture infectious dose (TCID_50_/mL) of SARS-CoV-2/Egypt/NRC-03/2020 isolate and incubated for 1 hr at 37°C. A total volume of 35 μl of the virus–sera mix was inoculated in duplicate to Vero-E6 cell in a 96-well tissue culture plates. After 1 hr of incubation at 37°C, the inoculums were removed. The plates were then incubated for three more days at 37°C in 5% CO_2_ in a humidified incubator. A virus back-titration was performed without immune serum to confirm TCID_50_ viral titer used. Cytopathic effect (CPE) was observed post 72 hrs of infection. The reciprocal of the serum dilution that protected cells from CPE was considered the nAb titer. Negative sera were given a value of 1:5.

### Statistical analysis

Data analysis was performed using SPSS v23 (IBM, Armonk, NY). Student’s t-test was used to compare means and chi-square to compare categories. A p-value ≤ 0.05 was considered statistically significant.

## Supporting information

S1 Table Distribution of demographic and health data of the study participants. (DOCX)

S2 Table Characteristics of seropositive infections at baseline. (DOCX)

S1 Fig Cumulative number of COVID-19 cases and deaths in Egypt. (DOCX)

S2 Fig Household transmission and seroprevalence of COVID-19 infection among close contact households. (DOCX)

S3 Fig Seroconversion neutralizing antibody titers among cohort participants, April to July 2020. (DOCX)

S4 Fig Sero-dynamics of COVID 19 antibodies in 200 subjects who tested positive in July and were resampled in October. (DOCX)
