# Supplementary material for: Incidence, household transmission, and neutralizing antibody seroprevalence of Coronavirus Disease 2019 in Egypt: Results of a community-based cohort
Source: PLoS Pathog. 2021 Mar 11;17(3):e1009413. doi: 10.1371/journal.ppat.1009413 (PMC7987187; doi:10.1371/journal.ppat.1009413)
Supplement: S1 Table — (DOCX) [file ppat.1009413.s001.docx]

**S1 Table. Distribution of demographic and health data of the study participants**

| Variable* | No. (%) |
| --- | --- |
| Age |  |
| 0-17 years | 762 (47.7) |
| 18-40 years | 516 (32.3) |
| 41-50 years | 156 (9.8) |
| 51-60 years | 101 (6.3) |
| 61-70 years | 47 (2.9) |
| 71 and above | 16 (1.0) |
| Sex |  |
| Female | 709 (44.4) |
| Male | 889 (55.6) |
| Educational level |  |
| Not educated | 523 (32.8) |
| Elementary | 551 (34.5) |
| Intermediate | 263 (16.5) |
| Vocational | 32 (2.0) |
| Secondary | 72 (4.5) |
| College | 83 (5.2) |
| Graduate degree | 72 (4.5) |
| Marital status |  |
| Divorced | 6 (0.4) |
| Married | 663 (41.5) |
| Single never married | 855 (53.5) |
| Widowed | 74 (4.6) |
| Occupation |  |
| Toddler | 243 (15.2) |
| Student | 553 (34.7) |
| Housewife | 437 (27.5) |
| Unskilled labor/Unemployed | 169 (10.6) |
| Skilled labor /professional | 192 (12.0) |
| Chronic disease |  |
| Yes | 159 (10.0) |
| No | 1439 (90.0) |
| Long-term breathing problems |  |
| Yes | 33 (2.1) |
| No | 1557 (97.9) |
| Current tobacco user |  |
| Yes | 115 (7.3) |
| No | 1456 (92.7) |

* Totals do not add up to 1598 for all variables due to missing data
