# Supplementary material for: Incidence, household transmission, and neutralizing antibody seroprevalence of Coronavirus Disease 2019 in Egypt: Results of a community-based cohort
Source: PLoS Pathog. 2021 Mar 11;17(3):e1009413. doi: 10.1371/journal.ppat.1009413 (PMC7987187; doi:10.1371/journal.ppat.1009413)
Supplement: S2 Table — (DOCX) [file ppat.1009413.s002.docx]

**S2 Table. Characteristics of seropositive infections at baseline**

| **Case type** | **Age** | **Gender** | **Symptoms** | **Positive RT-PCR** | **Titer at day 1** | **Titer at day 14** |
| --- | --- | --- | --- | --- | --- | --- |
| Index | 37 | Female | Yes | Yes | 40 | 320 |
| Index | 33 | Male | Yes | Yes | 20 | 40 |
| Index | 30 | Male | Yes | Yes | 80 | 160 |
| Contact | 45 | Female | Yes | Yes | 10 | 80 |
| Contact | 13 | Female | No | No | 160 | 160 |
| Contact | 39 | Female | No | Yes | 20 | 80 |
| Contact | 16 | Female | No | Yes | 40 | 20 |
| Contact | 66 | Male | No | Yes | 20 | 80 |
| Contact | 40 | Male | Yes | No | 320 | 160 |
| Contact | 20 | Female | Yes | Yes | 160 | 160 |
| Contact | 14 | Male | Yes | No | 320 | 160 |
| Contact | 11 | Female | Yes | No | 160 | 320 |
