# Supplementary material for: Incidence, household transmission, and neutralizing antibody seroprevalence of Coronavirus Disease 2019 in Egypt: Results of a community-based cohort
Source: PLoS Pathog. 2021 Mar 11;17(3):e1009413. doi: 10.1371/journal.ppat.1009413 (PMC7987187; doi:10.1371/journal.ppat.1009413)
Supplement: S3 Fig — (DOCX) [file ppat.1009413.s005.docx]

**S3 Fig. Seroconversion neutralizing antibody titers among cohort participants, April to July 2020**
